# Relationship of 5-HTTLPR Polymorphism with Various Factors of Pain Processing: Subjective Experience, Motor Responsiveness and Catastrophizing

**DOI:** 10.1371/journal.pone.0153089

**Published:** 2016-04-04

**Authors:** Miriam Kunz, Jürgen Hennig, Anna J. Karmann, Stefan Lautenbacher

**Affiliations:** 1 Department of General Practice, Geriatrics Section, University Medical Center Groningen, Groningen, The Netherlands; 2 Physiological Psychology, Department of Psychology, University of Bamberg, Bamberg, Germany; 3 Personality Psychology and Individual Differences, Department of Psychology, Justus-Liebig-University Giessen, Giessen, Germany; IIBB/CSIC/IDIBAPS, SPAIN

## Abstract

Although serotonin is known to play an important role in pain processing, the relationship between the polymorphism in 5-HTTLPR and pain processing is not well understood. To examine the relationship more comprehensively, various factors of pain processing having putative associations with 5-HT functioning were studied, namely the subjective pain experience (pain threshold, rating of experimental pain), catastrophizing about pain (Pain Catastrophizing Scale = PCS) and motor responsiveness (facial expression of pain). In 60 female and 67 male participants, heat pain stimuli were applied by a contact thermode to assess pain thresholds, supra-threshold ratings and a composite score of pain-relevant facial responses. Participants also completed the PCS and were grouped based on their 5-HTTLPR genotype (bi-allelic evaluation) into a group with s-allele carriers (ss, sl) and a second group without (ll). S-allele carriers proved to have lower pain thresholds and higher PCS scores. These two positive findings were unrelated to each other. No other difference between genotype groups became significant. In all analyses, “age” and “gender” were controlled for. In s-allele carriers the subjective pain experience and the tendency to catastrophize about pain was enhanced, suggesting that the s-allele might be a risk factor for the development and maintenance of pain. This risk factor seems to act via two independent routes, namely via the sensory processes of subjective pain experiences and via the booster effects of pain catastrophizing.

## Introduction

Serotonin (5-HT) has appeared to be involved in nociception and pain processing in multiple ways at peripheral and central levels [1]. Although a general link is by all means proven, it is much more difficult to tell exactly via which mechanisms and at which sites 5-HT unfolds its action. Molecular and systemic approaches have been developed to scrutinize the action of 5-HT. The systemic approaches are often based on the idea that in 5-HT synapses the availability of 5-HT determines its functionality, which is in turn dependent on the release, enzymatic cleavage and reuptake of 5-HT. Factors with great relevance for the functionality of 5-HT synapses are 5-HT transporters that recycle 5-HT into the presynaptic terminal. It depends on the genetic make-up how efficient these 5-HT transporters work. An intensively studied polymorphism is the serotonin transporter gene-linked polymorphic region (5-HTTLPR). A deletion/insertion in the 5-HTTLPR creates a short (s) allele and a long (l) allele (14- and 16-repeat alleles), which alters the promoter activity, with the short allele being associated with reduced transcriptional efficiency and thus, reduced 5-HT reuptake activity [2]. Systemic effects of these allele types have mainly been studied for psychiatric disorders such as anxiety and depression with mixed results [3].

There have also been a few attempts to relate the 5-HTTLPR polymorphism to pain processing. Most studies have focused on the subjective pain experience by assessing differences in pain thresholds. Whereas most of these studies found no effects of the 5-HTTLPR polymorphism on pain thresholds [4,5,6]; Hooten et al. [7] and Lindstedt et al. [8] observed that individuals with at least one short allele appeared to be less pain sensitive. However, the direct effects have appeared to be small and inconsistent.

A clearer picture of the effect of 5-HTTLPR polymorphism on pain processing might be obtained by not only assessing the subjective pain experience but by considering in addition various booster factors of pain processing that are also supposed to be 5-HT related. One such booster factor is the emotional appraisal of pain. The consequences of such emotional appraisal processes, like anxiety and depression, have repeatedly been shown to be related to 5-HTTLPR on the one hand [3] and have pain enhancing effects on the other hand [9]. According to such considerations, it is not surprising that chronic pain syndromes with a high emotional load have shown association with the short allele of 5-HTTLPR [10]. Thus, we decided to study pain catastrophizing as one booster factor of pain processing, given its relevance in emotional appraisal processes of pain and given its proven influence on pain processing (also in pain patients) [11].

Besides studying pain catastrophizing, we were also interested in a booster factor that might not appear compelling at first glance. Pain processing is also accompanied by motor responsiveness. This motor responsiveness is not only triggered by the intensity of pain but is also under the control of inhibitory regulation. We use the term “inhibition” to relate to the behavioral impulse control by prefrontal inhibitory circuits, which have appeared to be related 5-HT functioning [12], and not to the descending systems of pain inhibition. In line with this, Landro et al. [13] could demonstrate that persons with a short allele in 5-HTTLPR showed poorer performance in behavioral inhibition. This type of behavioral inhibition has been shown to be reflected in the facial expression of pain. It is known that we tend to inhibit our facial expressions of pain [14] and that facial stoicism (while experiencing pain) is due to strong inhibitory control of facial expressions and not a lack of subjective pain experience [15]. Interestingly, this behavioral inhibition of facial expression feeds back to influence the processing of pain [14].

In summary, we studied the association of 5-HTTLPR polymorphisms with (i) the subjective pain experience by assessing pain threshold and supra-threshold pain ratings and (ii) with two additional booster factors of pain processing, namely pain catastrophizing and motor responsiveness to pain (facial expression of pain) in a large sample of pain-free individuals. The three factors were methodologically designed to avoid any redundancy of variables and led to independent perspectives on pain processing. The pattern of associations should be informative about some of the mechanisms of 5-HT related actions on the pain systems.

## Materials and Methods

### Participants

127 healthy volunteers (female: N = 60, male: N = 67) between the ages of 18 and 65 years (mean age 36.3 years; SD = 14.9) participated in this study. Participants were recruited via advertisements in the local newspaper (Bamberg) and via advertisements posted at the campus of the University of Bamberg. Exclusion criteria were current experience of acute or chronic pain, psychological or physical illnesses and paresthesia or other types of somatosensory dysfunctions affecting the left lower leg (site of stimulation). Participants taking psychotropic drugs or analgesics were excluded from participation as well. All participants provided written informed consent and received monetary compensation. The study protocol (including the consent procedure) was approved by the Ethics committee of the University of Bamberg.

### Procedure

The experiment consisted of two testing sessions that were conducted on separate days. In the first session pain threshold, subjective and facial responses to heat pain as well as the degree of pain catastrophizing were assessed. In a second session (which took place several weeks till up to several months later) participants returned to the lab and bucca cells were sampled for 5-HTTLPR genotyping.

#### Pain induction and assessment of the subjective experience of pain

Thermal stimulation was applied on the outer part of the left lower leg by a Peltier based contact stimulation device (Medoc, TSA-2001, Ramat Yishai, Israel) with a 30 mm * 30 mm contact thermode.

Assessment of pain thresholds: The first session started with the assessment of individuals’ pain threshold. Heat pain thresholds were determined using the method of adjustment. Participants were asked to adjust a temperature starting from 38°C, using heating and cooling buttons, until they obtained a level which was barely painful. A constant press of the buttons produced a heating or cooling rate of 0.5°C/s. Following three familiarization trial, there were 4 trials and the average of these trials was used to constitute the threshold estimate.

Heat stimulation for assessing supra-threshold pain ratings (and facial expressions of pain): Following the assessment of pain thresholds, heat stimuli (5s (plateau); rate of change: 4°C/s; baseline temperature: 38°C; inter-stimulus-intervals of 15-20s) were applied to the lower leg. Two different stimulus intensities were applied, namely painful (+3°C above the pain threshold) as well as non-painful (-3°C below the pain threshold) intensities. Applying non-painful intensities allows determining which types of facial responses are indeed specific for painful experiences. Participants received 10 painful and 10 non-painful stimuli in a random order. The reason why we chose temperature intensities that were tailored to the individual pain threshold instead of fixed intensities was that we wanted to assess individual differences in supra-threshold pain ratings (and in facial expressiveness) that are not simply due to differences in pain threshold sensitivity.

Assessment of supra-threshold self-report ratings: Participants were asked to provide self-report ratings using an electronic visual analogue scale (VAS; 100mm) after each heat stimulus. The scale was labeled with a verbal anchor of ‘‘slightly painful” in the center so that all non-painful sensations should be rated below and all painful ones above. Participants were told that the left and right ends of the scale corresponded to “no sensation” and “extremely strong pain”, respectively. Participants had to rate the intensity of their non-painful and painful experiences by moving a cursor to the right or left and thereby choosing one location on the scale. Ratings had to be given within 10s after stimulus offset.

#### Assessing facial expressions of pain

The face of the participants was videotaped throughout the pain induction procedure. The camera was located approximately 1.0–2.0 m from the participant and participants were informed about the video recording. In order to enable offline segmentation of the videos, a LED visible to the camera, but not to the participant, was lit concurrently with the 5 s-thermal stimulation, starting when the target temperature had been reached. Participants were instructed to keep and upright position and not to talk during thermal stimulation

Facial expressions were coded from the video recordings using the Facial Action Coding System [16], which is based on anatomical analysis of facial movements and distinguishes 44 different Action Units (AUs) produced by single muscles or combinations of muscles. Four coders, trained by a certified FACS coder (qualified by passing an examination given by the developers of the system) identified the frequency and the intensity (5-point scale) of the different Action Units. Calculation of interrater reliability was based on 5% of the video recordings using the Ekman–Friesen formula [16] and lay between 0.82–0.87, which compares favourably with other research in the FACS literature. A software designed for the analysis of observational data (the Observer Video-Pro; Noldus Information Technology) was used to segment the videos and to enter the FACS codes into a time-related database. Time segments of 7 s beginning just after stimulus had reached the target temperature (5 seconds of plateau intensity + 2 seconds during temperature offset) were selected for scoring. In total 10 non-painful and 10 painful segments were analyzed in each participant. For the purpose of necessary data reduction, we combined those AUs that represent facial movements of similar muscles as has been done in preceding studies without any loss of information (e.g. Prkachin, 1992). Those combinations include AU1/2, AU6/7, AU9/10 and AU25/26/27. As was done in previous studies [14,17,18], pain-relevant AUs were selected using the following stepwise approach: (1) AUs had to occur in more than 5% of the painful segments recorded and (2) AUs had to be more frequent during pain than during non-painful segments (effect size d ≥0.5; these AUs are shaded in grey in Table 1). In the present study, brow lowering (contraction of the eyebrows), orbit tightening (contraction of the muscles surrounding the eyes), levator contraction (raising the upper lip and wrinkling the nose) and mouth opening (opening the mouth) proved to be pain-relevant AUs. Mean AU-frequency and mean AU-intensity values of these selected AUs were combined (product terms) to form a *composite score* of pain-relevant facial responses [17]. Due to the fact that these composite scores were not distributed normally, *square root transformed scores* were used for further analyses.

**Table 1:** Selection of pain-relevant facial responses: Facial Action Units (AUs) with a critical occurrence of more than 5% during painful stimulation are listed. Frequency of occurrence and effect sizes for frequency differences between “non-painful” and “painful” segments are given.

Action Unit	Description	Frequency of occurence	
		Percent^a^	*Effect size (Cohen’s d)*
AU1/2	brow raiser	12.2	*d = 0*.*29*
**AU4**	**brow lower**	**22.1**	***d = 0*.*69***
**AU6/7**	**orbit tightening**	**41.0**	***d = 0*.*70***
**AU9/10**	**levator contraction**	**14.7**	***d = 0*.*52***
AU14	dimpler	11.3	*d = 0*.*30*
**AU25/26/27**	**mouth opening**	**21.7**	***d = 0*.*52***

Medium and strong effect sizes (*d* ≥ 0.5) are marked in bold.

^a^ percent denotes the percentage of occurrence in the entire painful segments (10 painful heat stimuli).

#### Pain catastrophizing

A German translation of the Pain Catastrophizing Scale was used to assess catastrophic thinking related to pain (PCS [19,20]). Participants are instructed to reflect on thoughts or feelings during the past painful experiences. The scale contains 13 items that are rated on a five-point scale, with the end points ‘‘not at all” and ‘‘all the time”. For further analysis, the sum score was entered. The PCS has been widely used in research on pain catastrophizing, and has been shown to have high internal consistency. Pain catastrophizing is highly relevant in emotional appraisal of pain processes.

#### Genotyping

DNA was extracted from buccal cells using a standard commercial extraction kit (High Pure PCR Template Preparation Kit; Roche, Mannheim, Germany) in a MagNA Pure LC System (Roche). Genotyping for the classification of the 5-HTTLPR (to distinguish genotypes of s/s, s/l and l/l) was performed at the University of Giessen (Germany) as described previously by Alexander et al. [21]. Given that in previous studies the s-allele has been mostly linked to changes in pain sensitivity as well as to chronic pain conditions, we compared s-allele carriers (S/S, S/L) with individuals carrying no s-allele (L/L).

### Statistics

To test for effects of 5-HTTLPR genotype on pain thresholds and on pain catastrophizing, we conducted analysis of covariance (ANCOVA) with one between-subject factor “genotype” (s/s and s/l vs. no s-allele) and adding “age” and “gender” as covariates. The effects of 5-HTTLPR genotype on facial responses and pain ratings was assessed using ANCOVA with repeated measurement with one within “stimulus intensity” (non-painful vs. painful heat), one between-subject factor “genotype” ((s/s and s/l) vs. no s-allele) and adding “age” and “gender” as covariates.

Findings were considered to be statistically significant at α<0.05. SPSS-22 was used for all analyses.

## Results

### Genotypes–sample characteristics

Sample characteristics are depicted in Table 2. Groups separated by 5-HTTLPR genotype (s-allele carriers (s/l and s/s) versus no s-allele carriers (l/l)) did not differ significantly with respects to age and gender. Moreover, there was no significant deviation from Hardy—Weinberg-Equilibrium (x2 = 1.34 df = 1, p = 0.25).

**Table 2:** Demographic characteristics of participants and distribution of s-allele and no s-allele carriers in the present sample.

	LL	LS/SS	p
**N;** Hardy-Weinberg equilibrium (chi-square-test)	48	79 (65/14)	.25
**Age** (mean (SD)) (t-test)	35.7 (13.3)	36.6 (15.8)	.77
**Gender** (females/males) (chi-square test)	21/ 27	39/ 40	.54

### Subjective pain sensitivity

**Fig 1:**
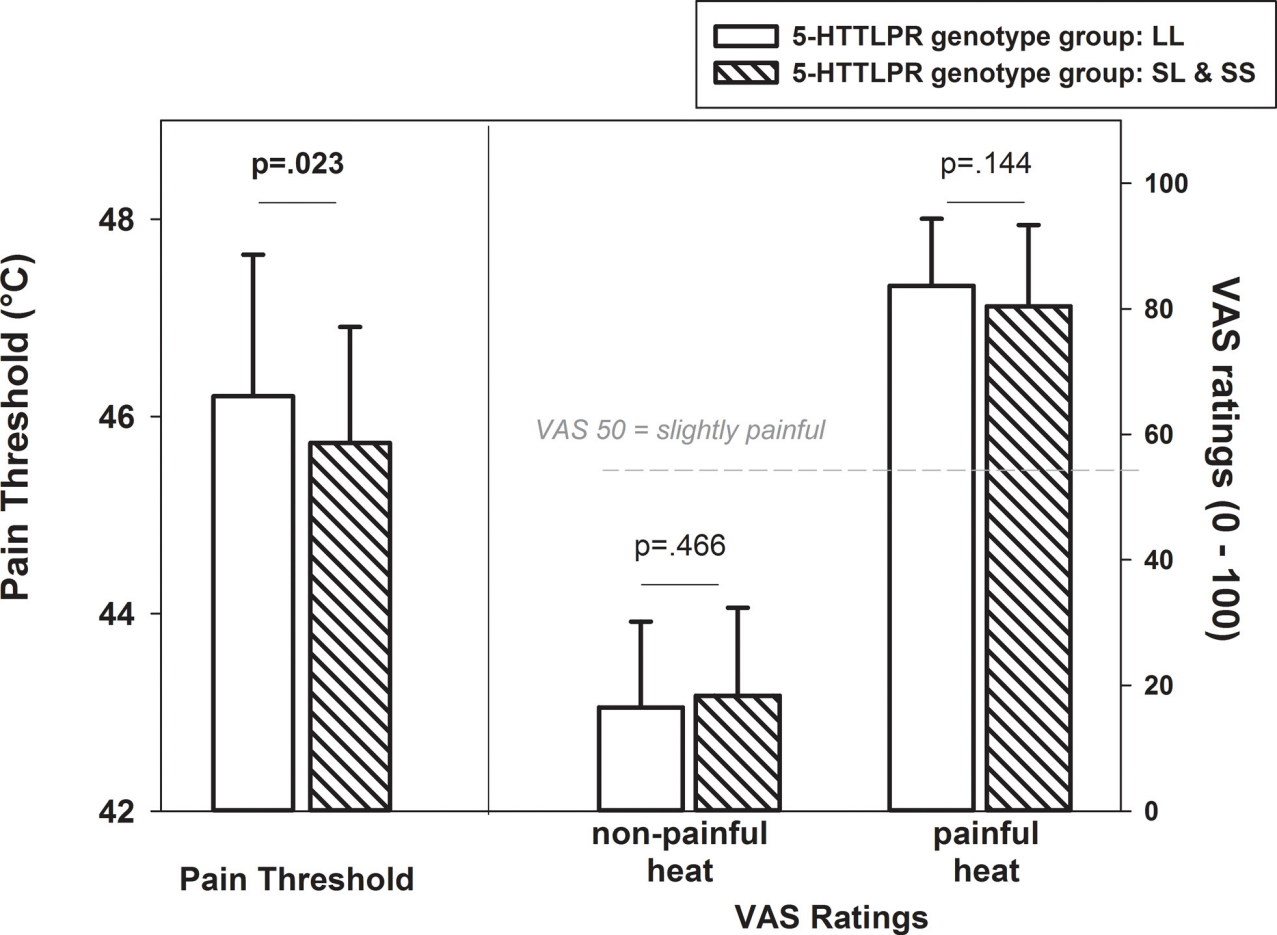
Pain thresholds and self-report ratings (VAS) (mean, SD) in s-allele and no s-allele carriers.

Pain thresholds differed significantly between groups with s-allele vs. no s-allele (F(1,123) = 4.53, p = 0.035), when adjusting for age and gender. As can be seen in Fig 1, s-allele carriers showed significantly reduced pain thresholds.

With regard to VAS ratings of the non-painful and supra-threshold painful heat stimuli, 5-HTTLPR genotype groups did not differ in their ratings (F(1,123) = 0.19, p = 0.663). As can be seen in Fig 1, painful stimuli were rated as significantly more painful compared to the non-painful heat stimuli (F(1,123) = 315.83, p<0.001). This was true for both s-allele as well as for no s-allele carriers, as indicated by a non-significant interaction between “genotype” and “stimulus intensity” (F(1,123) = 2.19, p = 0.142). Again, age and gender was controlled for.

### Facial expression

**Fig 2:**
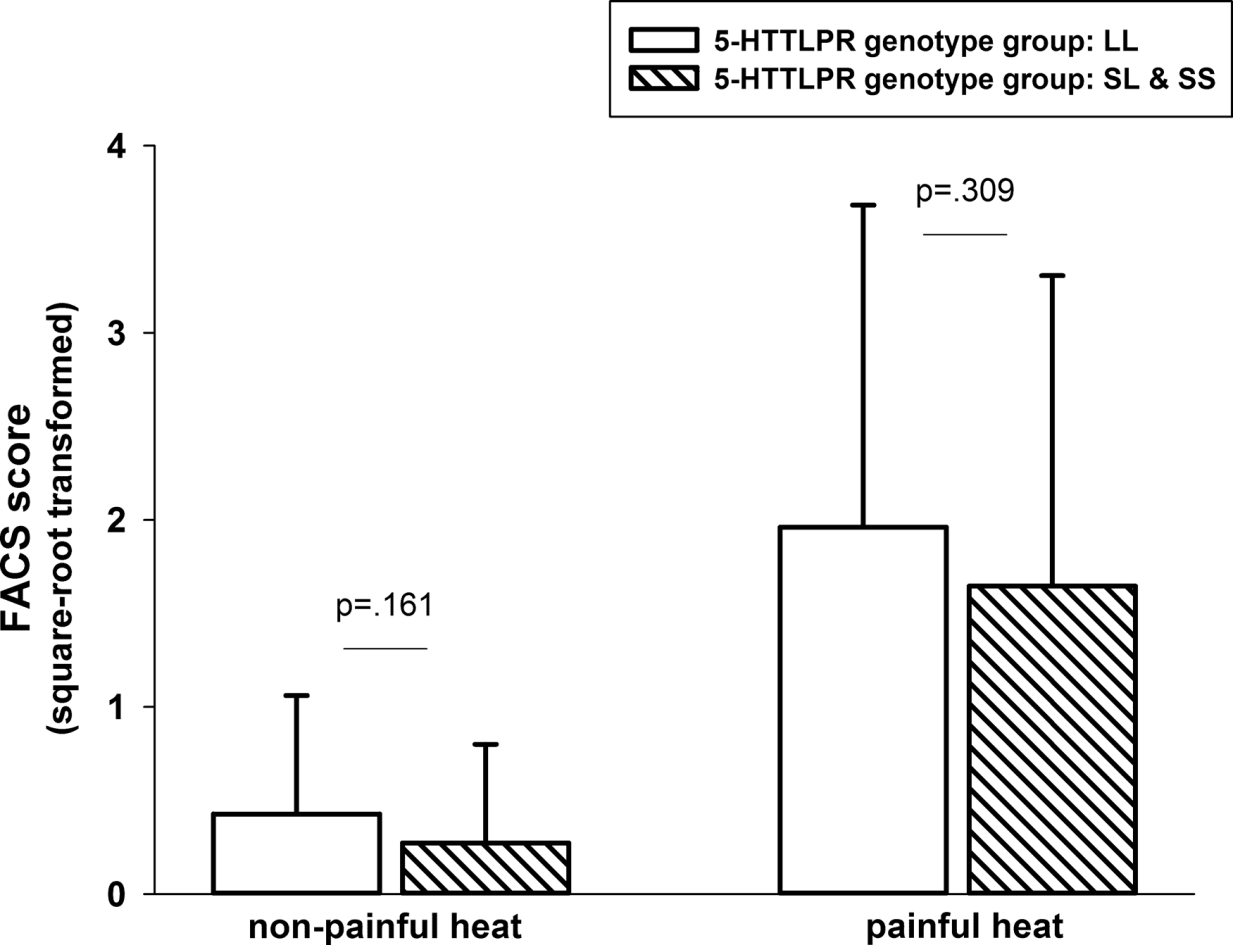
Facial expressions of pain (mean, SD) in response to non-painful and painful heat stimulation in s-allele and no s-allele carriers.

The degree of facial expressiveness did not differ between 5-HTTLPR genotype groups (F(1,123) = 2.01, p = 0.141). As can be seen in Fig 2, s-allele carriers were facially not more responsive to the thermal stimulation compared to the no s-allele carriers. As expected, there was a significant increase in facial expressions of pain from non-painful to painful heat stimulation (F(1,123) = 22.11, p<0.001). This increase in facial expressions of pain did not differ between s-allele vs. no s-allele carriers (F(1,123) = 0.36, p = 0.552). Again, age and gender was controlled for.

### Pain catastrophizing

**Fig 3:**
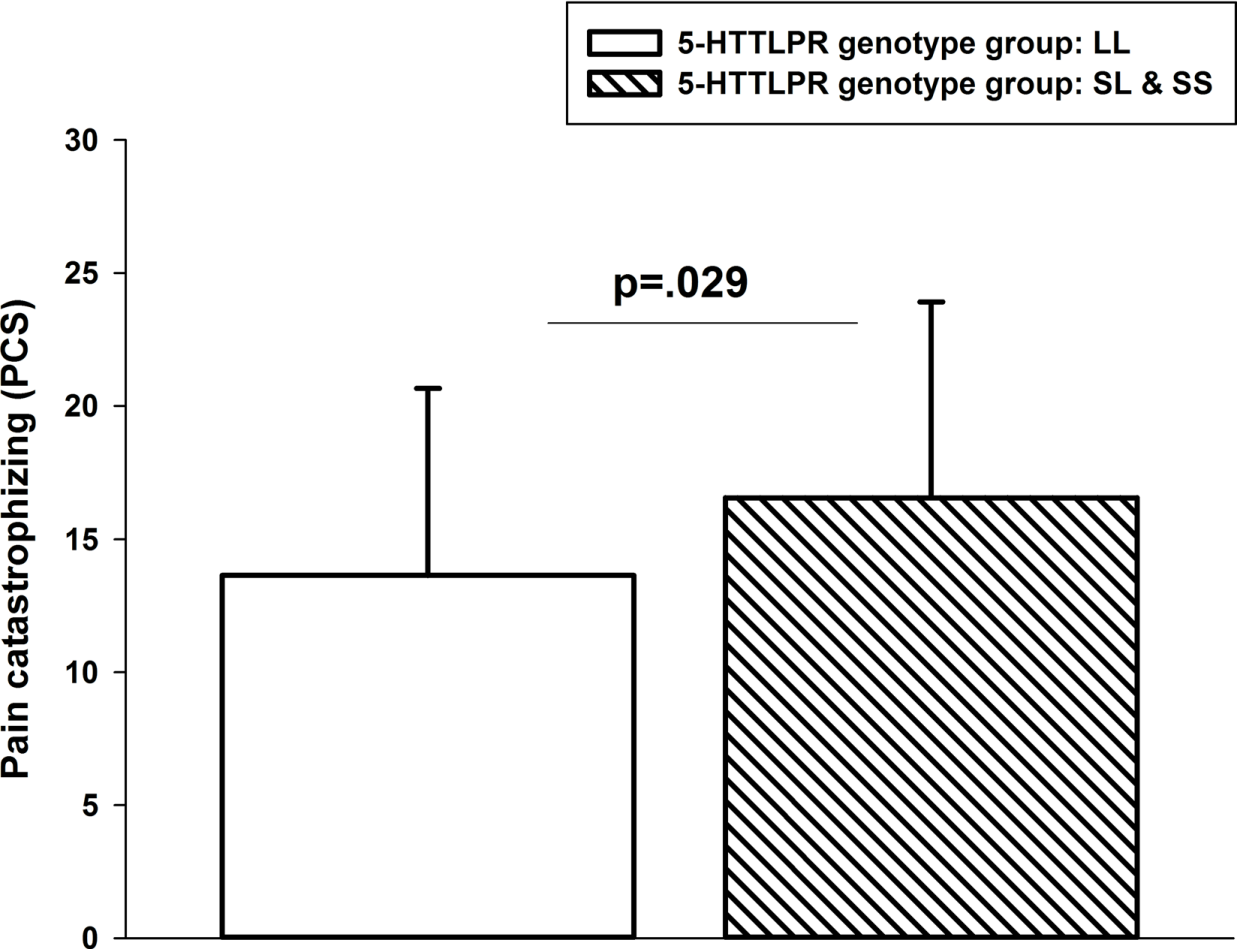
PCS scores (mean, SD) in s-allele and no s-allele carriers.

Groups with s-allele vs. no s-allele differed significantly in the degree to which they catastrophize about pain (F(1,123) = 4.89, p = 0.029), when adjusting for age and gender. As can be seen in Fig 3, s-allele carriers scored higher on the PCS scale compared to no s-allele carriers.

### Correlations between the various factors of pain processing

As stated in the introduction, we selected different factors of pain processing and wanted to avoid too strong redundancy between factors in order to allow independent perspectives on pain processing. In order to test this, we computed correlations between the pain variables and the results are given in Table 3. With the exception of a weak correlation between the two measures of subjective pain experience, namely pain threshold and supra-threshold pain ratings, none of the other pain variables correlated significantly.

**Table 3:** Correlations between the various factors of pain processing (r-values (p-values)).

	Pain Threshold	Supra-threshold pain ratings	Pain catastrophizing
Supra-threshold pain ratings	**r = .295 (p = .001)**		
Pain catastrophizing	r = -.007 (p = .940)	r = .050 (p = .577)	
Facial expression	r = .093 (p = .298)	r = .145 (p = .106)	r = .170 (p = .052)

Significant correlations are marked in bold.

## Discussion

The present study aimed at describing the association in a biallelic genetic evaluation between the polymorphism 5-HTTLPR and various factors of pain processing in pain-free individuals. Besides the subjective pain experience (represented by heat pain thresholds and heat pain ratings), two booster factors of pain processing were also studied, namely pain catastrophizing (questionnaire) and the facial expression of pain (FACS). These two booster factors were supposed to be indicative for the emotional appraisal of pain on the one hand and the behavioral inhibition of pain responsiveness on the other. The selected factors of pain processing have been shown to be related to 5-HT functioning in previous studies and showed to be largely independent from each other in the present study (as in earlier studies from our research group [17,22]). Carriers of at least one short (s) allele in 5-HTTLPR had lower heat pain thresholds and higher scores in the Pain Catastrophizing Scale (PCS) compared to participants with two long alleles. In contrast, no group differences were found in subjective and facial responses to painful heat stimulation that were tailored to the individual pain thresholds. The two positive findings were not related to each other, given the lack of a significant correlation between heat pain thresholds and pain catastrophizing scores.

Our finding of lower pain thresholds, indicating stronger subjective pain experience in s-allele individuals is not corroborated by earlier studies. There are some studies with no effect of the 5-HTTLPR polymorphism on pain thresholds [4,5,6]. In a study by Lindstedt et al. [8] individuals with at least one s-allele (S_A_/S_A_,L_G_/S_A (triallelic analyses)_) even had higher pain thresholds, which is opposite to our results. Hooten et al. [7] found the intermediate group (L_A_/L_G_,S_A_/L_G (triallelic analyses)_) to have the highest pain threshold. These earlier findings do not suggest that the s-allele necessarily reduces pain thresholds. Nevertheless, there is evidence that does converge with our findings of increased pain sensitivity in s-allele carriers. For example, observations in chronic pain patients have found that s-allele individuals are more prevalent in these conditions [10,23]. Moreover, healthy s-allele individuals were found to show reduced endogenous pain modulation (as assessed with the Conditioned Pain Modulation (CPM) paradigm, using a non-painful conditioning stimulus), which might render s-allele individuals more vulnerable to pain [24]. Furthermore, animal studies found heightened pain sensitivity in rats with low activity of the serotonin transporter [25], which converges with our findings of increased pain sensitivity in s-allele carriers. The lack of group differences in heat pain ratings does not refute the hypothesis of genotype-related differences in subjective pain experience because the intensity of supra-threshold heat stimuli was tailored to the individual heat pain threshold and thus, might have evened out inter-individual differences in subjective pain experience.

High scores in pain catastrophizing were also more frequent in individuals with at least one s-allele. As said, this finding does not simply replicate the result obtained when assessing pain thresholds because pain catastrophizing scores and pain thresholds were unrelated in the present study. Although several studies reported significant relations between pain catastrophizing and pain thresholds, the overall evidence is rather contradictory [26] with the degree of association often being only weak or nonexistent [17,22]. Interestingly, it seems to make a difference, whether one assesses dispositional pain catastrophizing (as we did) or situation-specific catastrophizing (catastrophic appraisal that only refers to the given noxious stimulation that one has just experienced). Whereas situation-specific catastrophizing does indeed show associations with pain thresholds, dispositional pain catastrophizing (that we assessed) does not [27]. Thus, finding no correlation between dispositional pain catastrophizing and pain thresholds is well in line with previous reports.

We cannot judge the reliability of our finding that s-allele individuals score higher on pain catastrophizing because our study is the first one to investigate this. Horjales-Araujo et al. [28] reported that a polymorphism in 5-HT receptor 3B (rs1176744) is associated with pain catastrophizing also using the Pain Catastrophizing Scale. This may suggest some relationship between 5-HT functioning and pain catastrophizing. However, the genotype analyzed by Horjales-Araujo et al. and the one analyzed in our study are of course not closely related in function. Nevertheless, the two findings may encourage further research on the relation between pain catastrophizing and 5-HT functioning, also given the rich literature about relationships between anxiety and 5-HT. Furthermore, a recent twin study found evidence for heritability of pain catastrophizing [29] and the 5-HTTLPR genotype might be an interesting gene candidate.

The 5-HTTLPR polymorphism did not show any association with our composite parameter of the facial expression of pain. We expected such an association because the facial expression of pain is not only driven by the intensity of pain, which might already be determined by 5-HT related mechanisms, but is also controlled by inhibitory gating of behavioral impulses [14,15]. We could recently provide evidence that the facial expression of pain is correlated with motor inhibitory control and supervised by frontostriatal circuits in the brain with inhibitory function [14,15]. Although motor impulse control appeared to be related in one study with the s-allele in 5-HTTLPR [13], our hypothesis of a relationship between the 5-HTTLPR polymorphism and the facial expression of pain could clearly not be verified. This does not, however, exclude that other 5-HT related mechanisms may play a role in the control of the facial expression of pain.

According to our data, s-allele carriers may experience more pain under conditions of a similar noxious load and may additionally and independently boost the processing of pain by catastrophic thinking. These two mechanisms of action may qualify the s-allele in 5-HTTLPR as a substantial risk factor for the development of pain problems. However, the effects were rather small and require replication before far-reaching conclusions can be drawn.

Some limitations ought to be mentioned. For studying genotype-phenotype associations and potential moderator effects of phenotypes amongst each other, which might have been considered for the present research questions, the sample size is still limited. However, the enormous investment of time and effort to FACS code the facial expression of pain will likely prevent larger samples even in future studies. Three domains of pain processing were in the focus of interest, namely subjective pain experience, emotional appraisal and motor/facial responsiveness. The number of measures used within each of these three domains, however, was rather limited. For example, we used pain intensities that were tailored to the individual pain thresholds and thus, the variability in pain ratings and in facial responses was artificially constricted, which might have impacted the genetic associations with these pain responses. Also, selecting only one pain modality limits the findings, since genetic associations often differ across modalities. Moreover, it would have been valuable to have also included measures of positive and negative mood, depression and/or anxiety, given the role of serotonin in mood and affective disorders [30, 31] and the seeming role of mood in pain processing. Thus, wider operationalization for each of the three domains (with additional measures) would have been preferable

In summary, the polymorphism 5-HTTLPR appeared to affect the subjective pain experience and the tendency to catastrophize about pain because s-allele carrier presented with lower pain thresholds and higher scores of pain catastrophizing, two variables that were unrelated in the present study. The facial expression of pain showed no association with 5-HT functioning as indicated by the present genetic evaluation. The present data suggest that the polymorphism 5-HTTLPR may affect the development and maintenance of pain via two independent routes, the sensory processes of subjective pain experiences and the booster effects of pain catastrophizing.

## Supporting Information

S1 File This file contains data (sample size, minimum, maximum, mean, standard deviation and variance) for pain threshold, pain catastrophizing, pain ratings and facial expression. The data are given separately for S-alleel carriers and non-S-allele carriers.
(PDF)
