# Supplementary material for: Relationship of 5-HTTLPR Polymorphism with Various Factors of Pain Processing: Subjective Experience, Motor Responsiveness and Catastrophizing
Source: PLoS One. 2016 Apr 4;11(4):e0153089. doi: 10.1371/journal.pone.0153089 (PMC4820275; doi:10.1371/journal.pone.0153089)
Supplement: S1 File — The data are given separately for S-alleel carriers and non-S-allele carriers. (PDF) [file pone.0153089.s001.pdf]

1. Pain Thresholds and 5HTTLPR

The tables below list pain threshold estimates that we assessed (N=sample size, Min=minimum, Max=maximum, mean, SD=standard deviation and variance).

Table 1 lists the values for the S-allele carriers. **Table 2** lists the values for the non S-allele carriers.

Table 1:

	N	Min	Max	Mean	SD	Variance
Pain Threshold	79	43.00	48.20	45.7314	1.17553	1.382

Table 2:

	N	Min	Max	Mean	SD	Variance
Pain Threshold	48	42.40	48.70	46.2063	1.43374	2.056

2. Pain Catastrophizing and 5HTTLPR

The tables below list pain catastrophizing scores (PCS) that we assessed (N=sample size, Min=minimum, Max=maximum, mean, SD=standard deviation and variance).

Table 3 lists the values for the S-allele carriers. **Table 4** lists the values for the non S-allele carriers.

Table 3

	N	Min	Max	Mean	SD	Variance
PCS_rumination	79	0	15	6.84	3.044	9.267
PCS_magnification	79	0	9	3.33	2.055	4.224
PCS_helplessness	79	0	15	6.41	3.716	13.808
PCS_SUM score	79	3.00	35.00	16.5696	7.34268	53.915

Table 4

	N	Min	Max	Mean	SD	Variance
PCS_rumination	48	0	14	5.60	3.413	11.648
PCS_magnification	48	0	7	2.85	2.042	4.170
PCS_helplessness	48	0	11	5.19	2.937	8.624
PCS_SUM score	48	.00	30.00	13.6458	7.01818	49.255

3. Pain Ratings and 5HTTLPR

The tables below list pain ratings (Visual Analog Scale (VAS)) that we assessed (N=sample size, Min=minimum, Max=maximum, mean, SD=standard deviation and variance).

Table 5 lists the values for the S-allele carriers. **Table 6** lists the values for the non S-allele carriers.

Table 5

	N	Min	Max	Mean	SD	Variance
VAS Rating non-painful heat	79	.00	54.70	18.3665	14.01085	196.304
VAS Rating painful heat	79	36.40	100.00	80.3921	12.94186	167.492

Table 6

	N	Minimum	Maximum	Mean	SD	Variance
VAS Rating non-painful heat	48	,00	56,70	16,5083	13,65739	186,524
VAS Rating painful heat	48	48,00	100,00	83,6583	10,66639	113,772

4. Facial expression and 5HTTLPR

The tables below list facial expressions (coded using the Facial Action Coding System) that we assessed (N=sample size, Min=minimum, Max=maximum, mean, SD=standard deviation and variance).

Table 7 lists the values for the S-allele carriers. **Table 8** lists the values for the non S-allele carriers.

Table 7

	N	Min	Max	Mean	SD	Variance
facial expression non-painful heat	78	.00	2.36	.2730	.52728	.278
facial expression painful heat	78	.00	6.38	1.6458	1.65993	2.755

Table 8

	N	Min	Max	Mean	SD	Variance
facial expression non-painful heat	48	.00	2.00	.4274	.63284	.400
facial expression painful heat	48	.00	6.71	1.9613	1.72164	2.964
